# Association of autosomal mosaic chromosomal alterations with risk of bladder cancer in Chinese adults: a prospective cohort study

**DOI:** 10.1038/s41419-024-07087-6

**Published:** 2024-09-30

**Authors:** Mingyu Song, Yuting Han, Yuxuan Zhao, Jun Lv, Canqing Yu, Pei Pei, Ling Yang, Iona Y. Millwood, Robin G. Walters, Yiping Chen, Huaidong Du, Xiaoming Yang, Wei Yao, Junshi Chen, Zhengming Chen, Giulio Genovese, Chikashi Terao, Liming Li, Dianjianyi Sun, China Kadoorie Biobank Collaborative Group, Mingyu Song, Yuting Han, Yuxuan Zhao, Jun Lv, Canqing Yu, Pei Pei, Ling Yang, Iona Y. Millwood, Robin G. Walters, Yiping Chen, Huaidong Du, Xiaoming Yang, Wei Yao, Junshi Chen, Zhengming Chen, Liming Li, Dianjianyi Sun

**Affiliations:** 1 Department of Epidemiology and Biostatistics, School of Public Health, Peking University, Beijing, 100191 China; 2 Peking University Center for Public Health and Epidemic Preparedness & Response, Beijing, 100191 China; 3 Key Laboratory of Epidemiology of Major Diseases (Peking University), Ministry of Education, Beijing, China; 4 Clinical Trial Service Unit & Epidemiological Studies Unit (CTSU), Nuffield Department of Population Health, University of Oxford, Oxford, United Kingdom; 5 NCDs Prevention and Control Department, Tongxiang CDC, Tongxiang, Zhejiang, China; 6 China National Center for Food Safety Risk Assessment, Beijing, China; 7 Program in Medical and Population Genetics, Broad Institute of MIT and Harvard, Cambridge, MA USA; 8 Stanley Center for Psychiatric Research, Broad Institute of MIT and Harvard, Cambridge, MA USA; 9 Department of Genetics, Harvard Medical School, Boston, MA USA; 10 Laboratory for Statistical and Translational Genetics, RIKEN Center for Integrative Medical Sciences, Kanagawa, Japan; 11 Clinical Research Center, Shizuoka General Hospital, Shizuoka, Japan; 12 The Department of Applied Genetics, The School of Pharmaceutical Sciences, University of Shizuoka, Shizuoka, Japan

**Keywords:** Predictive markers, Cancer epidemiology, Epidemiology, Cancer prevention, Bladder cancer

## Abstract

Little is known about the prospective association between autosomal mosaic chromosomal alterations (mCAs), a group of large-scale somatic mutations on autosomes, and bladder cancer. Here we utilized data from 99,877 participants who were free of physician-diagnosed cancer at baseline (2004–2008) of the China Kadoorie Biobank to estimate the associations between autosomal mCAs and bladder cancer (ICD-10: C67). A total of 2874 autosomal mCAs events among 2612 carriers (2.6%) were detected. After a median follow-up of 12.4 years, we discovered that participants with all autosomal mCAs exhibited higher risks of bladder cancer, with a multivariable-adjusted hazard ratio (HR) (95% confidence interval [CI]) of 2.60 (1.44, 4.70). The estimate of such association was even stronger for mosaic loss events (HR [95% CI]: 6.68 [2.92, 15.30]), while it was not significant for CN-LOH events. Both expanded (cell fraction ≥10%) and non-expanded autosomal mCAs, as well as mosaic loss, were associated with increased risks of bladder cancer. Of interest, physical activity (PA) significantly modified the associations of autosomal mCAs and mosaic loss (*P*_interaction_ = 0.038 and 0.012, respectively) with bladder cancer. The increased risks of bladder cancer were only observed with mCAs and mosaic loss among participants with a lower level of PA (HR [95% CI]: 5.11 [2.36, 11.09] and 16.30 [6.06, 43.81]), but not among participants with a higher level of PA. Our findings suggest that peripheral leukocyte autosomal mCAs may represent a novel risk factor for bladder cancer, and PA may serve as a potential intervention target for mCAs carriers.

## Introduction

Bladder cancer ranks as the 9th most commonly diagnosed malignant tumor worldwide [1]. In China, there were 92 883 new cases and 41 367 deaths of bladder cancer in 2022, according to the GLOBOCAN 2022, accounted for 15.1% and 18.8% of the global new cases and deaths, respectively, which ranked first in the world [1]. Bladder cancer causes a remarkable disease burden because of its high recurrence rate and the necessity for lifelong routine monitoring [2–4]. Prior studies have identified several risk factors for bladder cancer, such as tobacco smoking, occupational exposure [5, 6], genetic susceptibility [7], and age [8]. Such factors could increase the possibility of DNA impairment or influence cell cycle regulations, and eventually lead to somatic mutations, including point mutations, short indels, and mosaic chromosomal alterations (mCAs).

The mCAs refer to large-scale chromosomal rearrangements, including mosaic gain, mosaic loss, and copy-neutral loss of heterozygosity (CN-LOH), and are regarded as a genomic signature of accumulated DNA damage [9, 10]. It is well-established that mCAs on autosomal chromosomes are strongly associated with hematologic malignancy [11–17]. Although associations with other solid cancers, such as lung cancer, have been reported among individuals of European [11, 15] and Chinese ancestry [18], the evidence regarding the association between autosomal mCAs and bladder cancer is limited. Existing studies primarily originated from the Western population [11, 15, 17, 19], and only one study used a prospective design [15], limiting causal inference and the quantification of long-term risk of mCAs. Given the difference in genetic and environmental backgrounds, it remains unknown whether mCAs are associated with bladder cancer in the Chinese population and which subtypes exert the most detrimental effect.

Therefore, utilizing genotyping array data of Chinese adults from China Kadoorie Biobank (CKB), we aimed to estimate the prospective associations of autosomal mCAs and their subtypes with bladder cancer and to estimate the cumulative incidence of bladder cancer by mCAs status. Additionally, we sought to investigate potential effect modifiers of the association between mCAs and bladder cancer.

## Method

### Study population

The CKB study is a large-scale prospective cohort study of over 500,000 adults aged 30–79 years from 10 regions (5 urban and 5 rural) across China. Details of the study design have been described in previous papers [20, 21]. The baseline survey, conducted in 2004–2008, collected questionnaire data, physical measurements, and blood samples. The study was approved by the Ethical Review Committee of the Chinese Center for Disease Control and Prevention (Beijing, China) and the Oxford Tropical Research Ethics Committee, University of Oxford (Oxford, United Kingdom). Written informed consent forms from all participants were obtained.

### Genotyping and mCAs detection

Blood-derived DNA samples were genotyped by the Beijing Genomics Institute (Shenzhen, China) using two types of customized Affymetrix Axiom^®^ CKB arrays: Axiom CKB 1 (700 K markers in 33 K participants) and Axiom CKB 2 (800 K markers in 71 K participants). A total of 100 639 individuals with complete baseline data passed the quality control procedure, which was described previously [22]. The raw genotyping array intensity data were converted into VCF files using the Apt-probeset-genotype tool (v1.20.0) and BCFtools plugin affy2vcf (https://github.com/freeseek/gtc2vcf).

We followed the Mosaic Chromosomal Alterations (MoChA) pipeline [14, 23] (https://github.com/freeseek/mocha) to detect autosomal mCAs by utilizing the log R ratio (LRR) and B-allele frequency (BAF). Eagle2 (v2.4.1) (ref. [24]) was employed for long-range phasing. The highly polymorphic MHC (chr6: 27486711–33448264) and KIR (chr19: 54574747–55504099) regions were excluded from the mCAs calling. Additional filters were applied to exclude possible germline duplications and low-quality calls. Furthermore, we excluded participants who failed the sample quality control procedure (*n* = 342), as previously done [23]. We further excluded participants with physician-diagnosed cancer at baseline (*n* = 420), leaving 99,877 individuals in the current study.

The identified mCAs were classified into four categories: 1) mosaic loss; 2) mosaic gain; 3) CN-LOH, and 4) undetermined events, mCAs that could not be classified into any of the first three groups. Participants with any of the types mentioned above were regarded as carriers. To improve the power of the association analyses, we used the same strategy as Terao et al [13] that included undetermined “likely CN-LOH” (that was one telomere with |LRR | < 0.02) events into the CN-LOH group to analyze (*n* = 120). Cell fractions were estimated for each mCAs events based on subtypes and BAF deviations. According to the cell fraction, carriers of a specific mCA event could be categorized into non-expanded (cell fraction <10%) and expanded (cell fraction ≥10%) carriers. However, for some undetermined mCAs events, the cell fraction could not be estimated due to algorithmic issues.

### Ascertainment for incident bladder cancer events

Participants were followed up mainly through the linkage to the national health insurance database and local disease and death registry systems, which were annually supplemented with active follow-up. All events were coded according to the International Classification of Diseases, 10th Revision (ICD-10) by trained staff blinded to baseline information. In this study, participants were followed up from enrollment to the date of the bladder cancer diagnosis (ICD-10: C67), death, loss to follow-up, or Dec 31, 2018, whichever came first.

### Assessment of covariates

Demographic characteristics (sex, age, region, highest education, etc.), lifestyle factors in the past year (tobacco smoking, alcohol, tea consumption, and physical activity [PA], etc.), and medical and family history of cancer were all collected by trained health workers through a face-to-face electronic questionnaire. The daily level of PA was calculated by multiplying the metabolic equivalent tasks (METs) value for a particular type of activity by hours spent on that activity per day and summing up the MET-hours for all activities (including occupational, commuting, domestic, and leisure time activities) [25]. We defined the lower level of PA as those who engaged in a sex-specific lower 25% of total PA in the current study. Body height and weight were recorded by using calibrated instruments. All examinations were conducted following standard procedures. We calculated body mass index (BMI, kg/m^2^) by dividing weight by the square of height.

### Statistical analysis

Baseline characteristics by the status of all autosomal mCAs were presented as numbers (percentages) for categorical variables and means (standard deviations) for continuous variables. The distributions by the status of all autosomal mCAs were compared by using logistic regression adjusting for age, sex, and study regions, as appropriate.

Cox proportional hazard models were fitted to estimate the hazard ratios (HRs) and 95% confidence intervals (CIs) of mCAs with bladder cancer, with age as the time scale. The proportional hazard assumption for the Cox regression models was tested using the Schoenfeld residual, and no violation was discovered. Models were stratified by age in the 5-year interval and 10 study regions, and adjusted for potential confounders in the following steps. Model 1 included sex (men; women) and genotyping array (two types). Model 2 additionally included the top 5 principal components (continuous), family history of cancer (yes; no), and highest education (primary school and lower; middle or high school; and above the high school). Model 3 additionally included tobacco smoking (non-smoker; former smoker who had stopped for reasons other than illness; and current smoker or former smoker who had stopped because of illness [from one to 14 cigarettes or equivalent per day; 15–24 cigarettes or equivalent per day; and ≥25 cigarettes or equivalent per day]), alcohol consumption (never weekly drinker; former weekly drinker; weekly, but not daily drinker; 3 groups of daily drinker according to pure alcohol consumption per day [<15 g; 15–29 g; 30–59 g; and ≥60 g]), tea drinking (daily; not daily), BMI (continuous), and PA (low or high).

We analyzed all autosomal mCAs, mosaic loss, CN-LOH, and their subtypes categorized by cell fractions. For certain types of mCAs, we used participants without the corresponding type as a reference group. As for mosaic gain, we could not estimate its association with bladder cancer because there was no cases of bladder cancer among mosaic gain carriers. Sensitivity analysis was conducted by excluding participants who developed bladder cancer during the first two years of follow-up.

We further estimated the age-specific cumulative incidences of bladder cancer by the status of all autosomal mCAs and mosaic loss, as well as their corresponding joint categories with the level of PA. Cause-specific hazard models were fitted to account for the competing risk of death, with a model for bladder cancer and a model for death from other causes being separately developed. Both models were adjusted for the same set of covariates (as same as model 3) with all covariates being set to the average level of the study population.

Stratified analyses were performed according to sex, age, current smoking, and level of PA, respectively. Multiplicative interaction terms of each covariate and types of mCAs were included in the Cox regression model 3, and the likelihood-ratio tests were used to evaluate whether the model improved. For covariates with significant interaction with mCAs, we further estimated the joint effect of the corresponding covariate and mCAs on the risk of bladder cancer.

We used R software (v4.2.1) and Stata (v15.0) for statistical analyses and plots with the significance level of *P* < 0.05.

## Results

### Descriptive analysis

Of the 99,877 participants, the mean age at DNA collection was 53.7 ± 11.0 years, with 57.2% being women. A total of 2874 autosomal mCAs events were detected among 2612 carriers, with the prevalence was 2.6%. The majority of mCAs carriers (93.0%) had a single event, while 183 (7.0%) carried 2 to 16 events at different genomic positions (Supplementary Table 1). The CN-LOH comprised the largest proportion of detected mCAs events (62.0%), with the mosaic loss, mosaic gain, and undetermined events accounting for 17.4%, 7.7%, and 12.9%, respectively (Supplementary Table 2). The range of the mosaic cell fraction was 0.8%-77.4%, with more than half (52.2%) of autosomal mCAs carriers being the expanded mCAs (Supplementary Table 3).

The prevalence of autosomal mCAs increased with age in both sexes (*P* for trend <0.001). The prevalences of mCAs were 2.0 times (4.2% vs. 2.1%) higher in men and 1.7 times (3.4% vs. 2.0%) higher in women aged ≥65 years compared to those aged <45 years (Table 1, Supplementary Fig. 1). Also, autosomal mCAs carriers tended to have lower levels of physical activity, though the difference was marginally significant.

**Table 1:** Baseline characteristics of 99,877 participants by autosomal mCAs status.

	Autosomal mCAs carriers	Non-carriers	*P*
No. of participants	2612	97,265	–
Women	1407 (53.9)	55,765 (57.3)	0.012
Age group			
≤50	820 (31.4)	38,247 (39.3)	ref
50–60	746 (28.6)	29,797 (30.6)	0.003
>60	1046 (40.0)	29,221 (30.0)	<0.001
Urban area	1189 (45.5)	42,403 (43.6)	0.055
South area	1468 (56.2)	55,899 (57.5)	0.079
Primary school and lower	1467 (56.2)	51,576 (53.0)	0.527
Current smoker ^a^	856 (32.8)	30,287 (31.1)	0.567
Daily alcohol drinker	268 (10.3)	9289 (9.6)	0.997
Daily tea drinker	723 (27.7)	26,262 (27.0)	0.817
PA (MET-h/d)	18.2 ± 13.4	19.9 ± 13.8	0.046
BMI (kg/m^2^)	23.6 ± 3.5	23.7 ± 3.5	0.710
BMI (kg/m^2^) groups			
<18.5	152 (5.8)	5027 (5.2)	0.674
18.5–23.9	1316 (50.4)	49,373 (50.8)	ref
24.0–27.9	880 (33.7)	31,991 (32.9)	0.719
≥28.0	264 (10.1)	10,874 (11.2)	0.059
Family history of cancer	419 (16.0)	16,168 (16.6)	0.312

Categorical variables were presented as crude *n* (%), while continuous variables were presented as crude means ± SDs. All *P*-values were adjusted for age, sex and 10 study regions, as appropriate. ^a^Current-smoker included former smokers who had stopped smoking because of illness.

*mCAs* mosaic chromosomal alterations, *MET* metabolic equivalent of task, *PA* physical activity, *BMI* body mass index

### Association between autosomal mCAs and bladder cancer

**Fig. 1:**
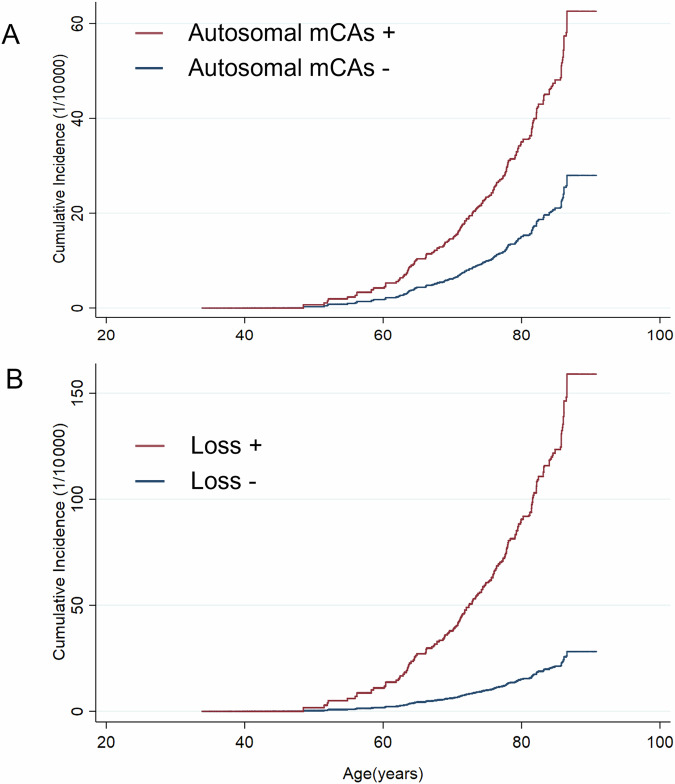
Cumulative incidence curves of bladder cancer by mCAs status. Cumulative incidence of bladder cancer for participants with or without autosomal mCAs (**A**) and mosaic loss (**B**). Cause-specific hazard models were used to account for the competing risk of death. Model for bladder cancer and model for death from other causes were separately developed, with the same set of variables (as same as model 3 in Table 2) included in both models. Cumulative incidences were estimated and plotted by the status of mCAs and mosaic loss, with all covariates being set to the average level of the study population. mCAs, mosaic chromosomal alterations.

During a median (interquartile range) follow-up of 12.4 (11.2–13.3) years, a total of 159 incident bladder cancer cases were identified among 99,877 individuals without physician-diagnosed cancer, with a crude incidence rate of 13.8 per 100,000 person-years. The distribution of the nature of mCAs in the participants with and without bladder cancer was described in Supplementary Fig. 2. Compared with their counterparts, participants with all autosomal mCAs and mosaic loss events had higher risks of incident bladder cancer, with the multivariable-adjusted HRs (95% CIs) of 2.60 (1.44, 4.70) and 6.68 (2.92, 15.30), respectively (Table 2). No significant association was observed for CN-LOH events. The cumulative incidence of autosomal mCAs carriers and mosaic loss carriers increased more rapidly with age in comparison to non-carriers, especially for loss carriers (Fig. 1). In terms of cell fraction analyses for autosomal mCAs and loss, both expanded and non-expanded mCAs were associated with higher risks of bladder cancer. However, we only observed a significant association between non-expanded CN-LOH and bladder cancer.

**Table 2:** Associations of all autosomal mCAs and mCAs subtypes with bladder cancer among 99,877 participants.

			HR (95% CI)		
	Incident cases/N	Incidence rate (1/100 000)	Model 1	Model 2	Model 3
All autosomal mCAs -	147/97,265	13.1	1.00	1.00	1.00
All autosomal mCAs +	12/2612	41.1	2.60 (1.44, 4.69)	2.60 (1.44, 4.70)	2.60 (1.44, 4.70)
Non-expanded	6/1270	42.8	2.63 (1.16, 5.96)	2.64 (1.16, 6.00)	2.60 (1.14, 5.92)
Expanded	6/1342	39.5	2.57 (1.13, 5.83)	2.55 (1.13, 5.80)	2.59 (1.14, 5.89)
**mCAs subtypes**					
Loss -	153/99,454	13.3	1.00	1.00	1.00
Loss +	6/423	133.6	6.75 (2.96, 15.37)	6.70 (2.93, 15.30)	6.68 (2.92, 15.30)
Non-expanded	1/61	148.9	7.78 (1.07, 56.38)	8.11 (1.12, 58.89)	8.96 (1.23, 65.28)
Expanded	5/362	130.9	6.57 (2.68, 16.14)	6.47 (2.63, 15.94)	6.35 (2.57, 15.70)
CN-LOH -	154/98,162	13.6	1.00	1.00	1.00
CN-LOH +	5/1715	25.8	1.73 (0.71, 4.24)	1.74 (0.71, 4.25)	1.76 (0.72, 4.30)
Non-expanded	4/789	45.5	2.87 (1.06, 7.78)	2.89 (1.07, 7.83)	2.83 (1.04, 7.67)
Expanded	1/926	9.5	0.67 (0.09, 4.80)	0.67 (0.09, 4.80)	0.70 (0.10, 5.02)

There were no bladder cancer cases among mosaic gain carriers. All multivariate models were stratified by age in the 5-year interval and 10 regions, and adjusted for sex and genotyping array (model 1), top 5 principal components, family history of cancer, and highest education (model 2), tobacco smoking, alcohol consumption, tea drinking, BMI, and PA (model 3).

*HR* hazard ratios, *CI* confidence interval, *mCAs* mosaic chromosomal alterations, *CN-LOH* copy-neutral loss of heterozygosity, *BMI* body mass index, *PA* physical activity

After excluding the bladder cancer diagnosed within the first two years of follow-up, the associations were not materially altered (Supplementary Table 4).

### Modifying effects of physical activity

**Fig. 2:**
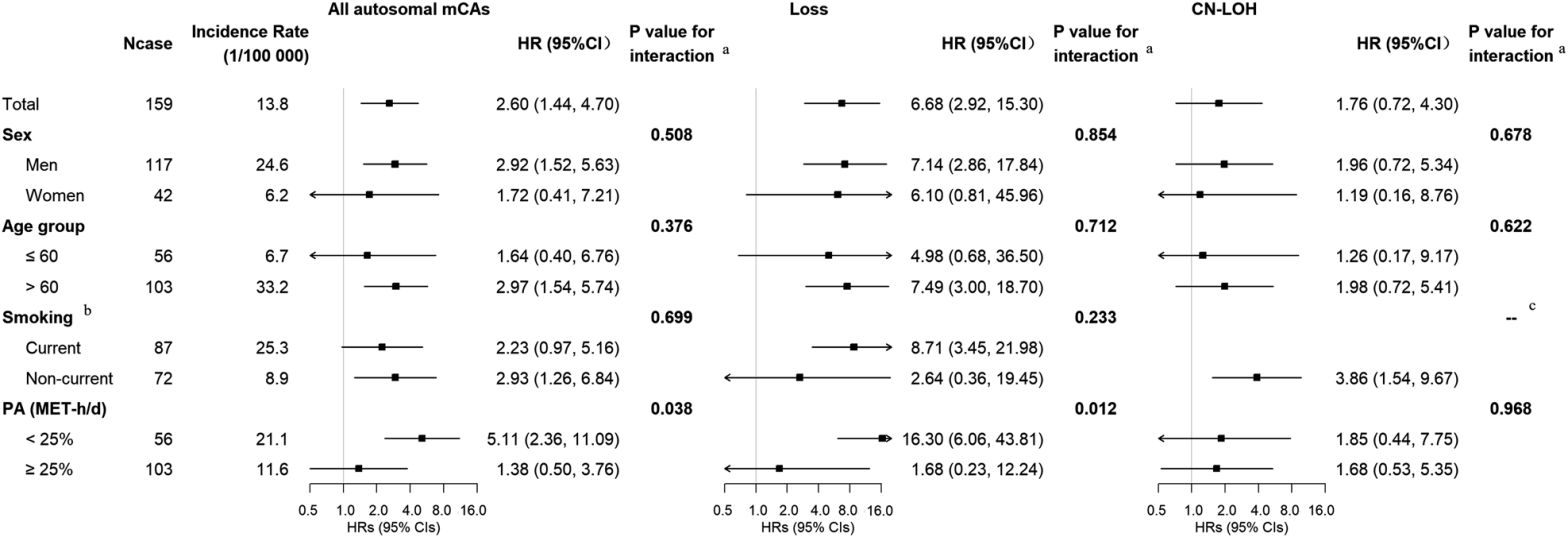
Associations of all autosomal mCAs and mCAs subtypes with bladder cancer by baseline subgroups. Adjusted covariates in the models were consistent with model 3 in Table 2, as appropriate. ^a^The *P* values for interaction were computed with likelihood-ratio tests comparing models with and without multiplicative interaction terms of the baseline stratifying variable and types of mCAs. ^b^Current-smoker included former smokers who had stopped smoking because of illness. ^c^There were no bladder cancer cases among current smokers with CN-LOH. HR hazard ratios, CI confidence interval, mCAs mosaic chromosomal alterations, CN-LOH copy-neutral loss of heterozygosity, PA physical activity, MET metabolic equivalent of task.

**Fig. 3:**
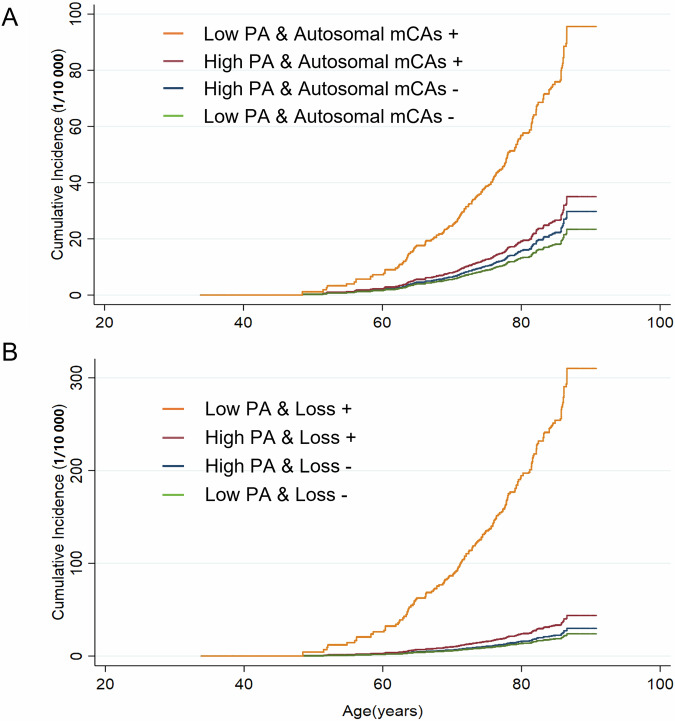
Cumulative incidence curves of bladder cancer by mCAs status and physical activity levels. Cumulative incidence of bladder cancer according to joint categories of level of physical activities and autosomal mCAs (**A**) or mosaic loss (**B**). Cause-specific hazard models were used to account for the competing risk of death. Model for bladder cancer and model for death from other causes were separately developed, with the same set of variables (as same as model 3 in Table 2) included in both models. Cumulative incidences were estimated and plotted by joint categories of level of PA (classified into low and high groups by the 25^th^ percentile of sex-specific MET-h per day) and autosomal mCAs or mosaic loss, with all covariates being set to the average level of the study population. PA physical activity, mCAs mosaic chromosomal alterations, MET metabolic equivalent of task.

In the stratified analysis, there were no significant interactions between all autosomal mCAs, mCAs subtypes and age, sex, or smoking with respect to bladder cancer. (Fig. 2). We found that PA significantly modified the associations of autosomal mCAs and mosaic loss (*P*_interaction_ = 0.038 and 0.012, respectively) with bladder cancer (Fig. 2). Among participants with a lower level of PA, autosomal mCAs were strongly associated with an increased risk of bladder cancer, with an HR (95% CI) was 5.11 (2.36, 11.09). The same situation came to mosaic loss with an even higher HR (95% CI) of 16.30 (6.06, 43.81). Whereas, such associations were not observed among participants with a higher level of PA. Joint analyses revealed that autosomal mCAs and mosaic loss carriers with a lower level of PA (Low PA & Autosomal mCAs + / Loss +) had the highest cumulative incidences of bladder cancer after the age of around 50 years (Fig. 3). Compared with High PA & Autosomal mCAs - / Loss - groups, the HRs (95% CIs) were 4.43 (2.10, 9.36) for Low PA & Autosomal mCAs + group and 16.12 (6.34, 40.98) for Low PA & Loss + group, respectively (Supplementary Table 5).

## Discussion

In this prospective study of approximately 100,000 Chinese adults aged 30–79 years, we found that peripheral blood autosomal mCAs were significantly associated with an increased risk of bladder cancer. Such association was mainly driven by mosaic loss events. Furthermore, we found statistically significant interaction between PA and mCAs on bladder cancer. The associations of autosomal mCAs and loss with bladder cancer were only observed among participants with a lower level of PA.

The current study found a significant association between autosomal mCAs and bladder cancer in the Chinese population, which was not reported by previous studies primarily based on Western populations [11, 15, 17, 19]. A pooled analysis of individual data from a large set of Genome-Wide Association Studies (GWAS) involving 57 853 individuals [11] and a subsequent study with a larger sample [19] (*n* = 127 179) did not find a significant association between autosomal mCAs and prevalent bladder cancer, with the similar odds ratios (95% CI) of 1.30 (0.90, 1.89) and 1.29 (0.90, 1.85), respectively. Another two studies based on the UK Biobank [15, 17], one of which explored the prospective association of expanded autosomal mCAs with incident bladder cancer and did not report an increased risk either [15]. Distinct findings may indicate the ancestry-specific effect of mCAs on bladder cancer. The positive association could be explained by some underlying mechanisms. One potential mechanism could be that the mutated segments cover specific bladder cancer-related pathogenic genes, such as *TP73* [26], *KMT2C* [27, 28], and *FOXA2* [29], or affect some tumor suppressor genes. For instance, the deletion or inactivation of *NF1* can disrupt normal cell cycle regulation [30], which might contribute to the development of bladder cancer in some autosomal mCAs carriers. In addition, hematopoietic stem cells and peripheral blood leukocytes play a crucial role in the immune system. The occurrence of mCAs in leukocytes may disrupt immune homeostasis and weaken defense against infectious diseases, including genitourinary infections [15], which may increase the risk of bladder cancer [31, 32]. Our findings indicate that identifying mCAs carriers may serve as an independent predictor of bladder cancer to facilitate the early detection of high-risk groups for bladder cancer.

Additionally, our data further suggested that mosaic loss events might be the main contributor to the increased risk of bladder cancer in autosomal mCAs carriers. Although this finding has not been reported in prior studies, loss events have been observed as a major driver of high risk in other adverse health outcomes. For instance, a recent study conducted in the Nanjing Lung Cancer Cohort of China and the UK Biobank population (*n* = 461,069) demonstrated that the effect of mCAs on lung cancer mainly came from loss events [18]. Furthermore, an earlier study in the UK Biobank also observed that loss carriers had the highest mortality risk among cancer patients [23]. Therefore, subtype-specific effects of mCAs on health outcomes probably exist and loss is more likely to be the most hazardous type. Also, there were studies indicating that the risk of hematologic malignancy and lung cancer increased with increasing cell fraction [15, 18]. Our study found that expanded and non-expanded mCA carriers had a similarly increased risk of bladder cancer. Supposing the dose-response effects of cell fraction on adverse health outcomes exist, one possible explanation could be that mCAs carriers with the high cell fraction might have been diagnosed with cancer, in poor health, or even dead before cohort enrollment, resulting in not being included in the current study. However, evidence of the cell fraction-dependent effect of mCAs is still limited, especially regarding other non-hematologic cancers. We cannot preclude the possibility that the effects of cell fraction differ across different diseases.

Interestingly, we found that the associations of autosomal mCAs and mosaic loss with bladder cancer were only evident among those with a lower level of PA (*P* for interaction <0.05). Although these results would be considered exploratory, the interactions between PA and mCAs with the risk of bladder cancer may be biologically plausible. On the one hand, longitudinal analyses of individuals with chromosomal structural aberrations suggested that aberrant cells could naturally self-removal from peripheral blood [19, 33]. And, previous studies have shown that PA had a positive effect on antioxidant capacity, reduced DNA damage, and promoted DNA self-repair processes [34, 35]. Hence, the interaction may be explained by a reversal of the mCAs status resulting from PA. In the current study, we observed a marginally significant association between PA and all autosomal mCAs (Table 1), which could partially support the above hypothesis. On the other hand, PA has the potential to block the pathogenesis of mCAs. Previous studies indicated that the pathogenesis of mCAs is related to immune impairment and the inflammatory response [36], both of which could be positively affected by PA [37]. Although the mechanisms remained unclear, the findings emphasized the health benefits of encouraging PA and indicated a potential role of PA in preventing bladder cancer among mCAs carriers in China.

To the best of our knowledge, the present study was the first and the largest autosomal mCAs-related study conducted in a community-based population in China. Given the prospective cohort design after a median follow-up over 10 years, our study was less prone to reverse causation compared with previous studies [11, 17, 19]. Furthermore, our study comprehensively controlled for known and possible confounders for the association estimates. Nevertheless, our study should be interpreted in light of several limitations. First, mCAs events in this study were only measured at baseline, though it could dynamically progress with age. Therefore, the actual association of mCAs with bladder cancer might be underestimated. In addition, our study also did not consider changes in PA during follow-up. However, our previous study pointed out that most participants in CKB kept their lifestyles during the follow-up [38]. Second, even though we had excluded cancer patients at baseline, there was still the possibility of undiagnosed bladder cancer in our sample due to the long asymptomatic duration. However, we excluded participants who developed bladder cancer during the first two years of follow-up in sensitivity analysis and the results remained robust. Third, although the sample size was a strength, the mCAs events and bladder cancer cases identified in this study were still limited. The primary findings, however, should be reliable because the estimates for the associations of autosomal mCAs and loss with bladder cancer were large, even with comprehensive adjustment. Nevertheless, further validation with a larger sample size, especially of findings from subtype-specific analyses and stratified analyses, should be conducted by other studies.

In summary, our results indicated that peripheral blood leukocyte autosomal mCAs carriers were at a significantly greater risk of incident bladder cancer, especially for mosaic loss carriers. Additionally, the positive associations of mCAs with bladder cancer were only observed among participants with lower levels of PA. Our findings indicate autosomal mCAs as a novel risk factor for bladder cancer and PA as a potential intervention target for mCAs carriers in China to reduce the risk of bladder cancer. With the decreasing cost of genotyping and advanced detection techniques, future studies are expected to examine the association between autosomal mCAs and bladder cancer in multiethnic populations with larger sample sizes. Furthermore, single autosome-level analyses should be conducted to gain insight into the mechanism of associations between mCAs and bladder cancer.

## Supplementary information

supplemental tables and figures

## Data Availability

Data from baseline, first and second resurveys, and disease follow-up are available under the CKB Open Access Data Policy to bona fide researchers. Full details of the CKB Data Sharing Policy are available at www.ckbiobank.org.

## Code availability

Software programs used in this study are all publicly available. Detection and classification of autosomal mCAs were performed using the Mosaic Chromosomal Alterations (MoChA) pipeline (https://github.com/freeseek/mocha). R (v4.2.1) can be assessed from the CRAN website (https://cran.r-project.org/). Stata (v15.0) can be assessed from STATA website (https://www.stata.com/stata15/). Additional analysis codes can be accessed by contacting the corresponding author for research purposes.
