# Supplementary material for: Association of autosomal mosaic chromosomal alterations with risk of bladder cancer in Chinese adults: a prospective cohort study
Source: Cell Death Dis. 2024 Sep 30;15(9):706. doi: 10.1038/s41419-024-07087-6 (PMC11443067; doi:10.1038/s41419-024-07087-6)
Supplement: Supplementary file 1 — supplemental tables and figures [file 41419_2024_7087_MOESM1_ESM.docx]

**Supplementary material for: “Association of autosomal mosaic chromosomal alterations with risk of bladder cancer in Chinese adults: a prospective cohort study”**

**Contents:**

[Members of the China Kadoorie Biobank Collaborative Group 2](#_Toc173332069)

[Supplementary Table 1: Number of autosomal mCAs events per individual among 99,877 participants. 4](#_Toc173332070)

[Supplementary Table 2: Chromosomal distribution of detected mCAs among 99,877 participants by event types. 5](#_Toc173332071)

[Supplementary Table 3: Counts of mCAs and expanded mCAs by event type of all carriers. 6](#_Toc173332072)

[Supplementary Table 4: Associations of all autosomal mCAs and mCAs subtypes with bladder cancer after excluding the bladder cancer identified in the first two years of follow-up. 7](#_Toc173332073)

[Supplementary Table 5: The HRs and 95%CIs of bladder cancer according to joint categories of level of physical activity and autosomal mCAs or mosaic loss. 8](#_Toc173332074)

[Supplementary Figure 1：Association between age and the status of autosomal mCAs by sex among CKB population. 9](#_Toc173332075)

[Supplementary Figure 2：Distribution of the nature of mCAs in the participants with and without bladder cancer. 10](#_Toc173332076)

Members of the China Kadoorie Biobank Collaborative Group

**International Steering Committee:** Junshi Chen, Zhengming Chen (PI), Robert Clarke, Rory Collins, Yu Guo, Liming Li (PI), Jun Lv, Richard Peto, Robin Walters. **International Co-ordinating Centre, Oxford:** Daniel Avery, Derrick Bennett, Ruth Boxall, Sue Burgess, Ka Hung Chan, Yumei Chang, Yiping Chen, Zhengming Chen, Johnathan Clarke; Robert Clarke, Huaidong Du, Ahmed Edris Mohamed, Zammy Fairhurst-Hunter, Hannah Fry, Simon Gilbert, Alex Hacker, Mike Hill, Michael Holmes, Pek Kei Im, Andri Iona, Maria Kakkoura, Christiana Kartsonaki, Rene Kerosi, Kuang Lin, Mohsen Mazidi, Iona Millwood, Sam Morris, Qunhua Nie, Alfred Pozarickij, Paul Ryder, Saredo Said, Sam Sansome, Dan Schmidt, Paul Sherliker, Rajani Sohoni, Becky Stevens, Iain Turnbull, Robin Walters, Lin Wang, Neil Wright, Ling Yang, Xiaoming Yang, Pang Yao.

**National Co-ordinating Centre, Beijing:** Dianjianyi Sun, Yu Guo, Xiao Han, Can Hou, Jun Lv, Pei Pei, Chao Liu, Canqing Yu, Qingmei Xia, Lang Pan.

**10 Regional Co-ordinating Centres: Qingdao** CDC: Zengchang Pang, Ruqin Gao, Shanpeng Li, Haiping Duan, Shaojie Wang, Yongmei Liu, Ranran Du, Liang Cheng, Xiaocao Tian, Hua Zhang, Yaoming Zhai, Feng Ning, Xiaohui Sun, Feifei Li. Licang CDC: Silu Lv, Junzheng Wang, Wei Hou, Xiaoyan Zheng, **Heilongjiang** Provincial CDC: Wei Sun, Shichun Yan, Xiaoming Cui. Nangang CDC: Chi Wang, Zhenyuan Wu, Lishun Zhai, Yanjie Li, Zhaoxi Pang, Shiwen Dong. **Hainan** Provincial CDC: Huiming Luo, Yan Chen, Xingren Wang, Dingwei Sun, Tingting Ou. Meilan CDC: Xiangyang Zheng, Zhendong Guo, Shukuan Wu, Yilei Li, Lihui Li. **Jiangsu** Provincial CDC: Ming Wu, Yonglin Zhou, Jinyi Zhou, Ran Tao, Jian Su, Xikang Fan. Suzhou CDC: Fang Liu, Jun Zhang, Yan Lu, Li Xing, Yujie Hua, Jianrong Jin, Jingchao Liu. **Guangxi** Provincial CDC: Ge Zhong, Xianming Liao, Wei Mao,Zhenzhen Lu. Liuzhou CDC: Lifang Zhou, Zhongshu Qin, Jian Lan, Tingping Zhu, Liuping Wei, Liyuan Zhou, Sisi Wang. **Sichuan** Provincial CDC: Xianping Wu, Ningmei Zhang, Xiaofang Chen, Xiaoyu Chang. Pengzhou CDC: Mingqiang Yuan, Xia Wu, Xiaofang Chen, Wei Jiang, Jiaqiu Liu, Qiang Sun. **Gansu** Provincial CDC: Faqing Chen, Hupeng He, Xiaolan Ren. Maiji CDC: Zhongyi Hui, Jianjun Feng, Weijie Hu, Peng Wen, Yalin Chen, Fei Wang , Xiaofang Zhang. **Henan** Provincial CDC: Yibin Hao, Zhiwei Han, Kai Kang, Shixian Feng, Huizi Tian. Huixian CDC: Xiaolin Li, Huarong Sun, Pan He, Chen Hu, Xukui Zhang. **Zhejiang** Provincial CDC: Min Yu, Ruying Hu, Hao Wang, Weiwei Gong, Meng Wang. Tongxiang CDC: Huanxu Zhang, Yuan Cao, Kaixu Xie, Lingli Chen, Xiaomei Tu. **Hunan** Provincial CDC: Xiaojun Li, Li Yin, Huilin Liu, Donghui Jin. Liuyang CDC: Xin Xu, Jianwei Chen, Yuan Peng, Chan Qu, Libo Zhang.

Supplementary Table 1: Number of autosomal mCAs events per individual among 99,877 participants.

| **No. of events** | **No. of individuals** |
| --- | --- |
| 0 | 97,265 |
| 1 | 2,429 |
| 2 | 147 |
| 3 | 23 |
| 4 | 8 |
| 6 | 2 |
| 10 | 1 |
| 12 | 1 |
| 16 | 1 |

mCAs, mosaic chromosomal alterations

Supplementary Table 2: Chromosomal distribution of detected mCAs among 99,877 participants by event types.

|  | **CN-LOH** | **Gain** | **Loss** | **Undetermined** | **Total** |
| --- | --- | --- | --- | --- | --- |
| Chr1 | 194 | 21 | 7 | 26 | 248 |
| Chr2 | 61 | 9 | 32 | 12 | 114 |
| Chr3 | 122 | 18 | 4 | 14 | 158 |
| Chr4 | 72 | 10 | 41 | 47 | 170 |
| Chr5 | 48 | 16 | 26 | 12 | 102 |
| Chr6 | 81 | 9 | 22 | 11 | 123 |
| Chr7 | 68 | 3 | 18 | 15 | 104 |
| Chr8 | 58 | 13 | 13 | 34 | 118 |
| Chr9 | 128 | 10 | 9 | 7 | 154 |
| Chr10 | 104 | 2 | 45 | 29 | 180 |
| Chr11 | 119 | 3 | 37 | 14 | 173 |
| Chr12 | 54 | 27 | 12 | 21 | 114 |
| Chr13 | 56 | 3 | 28 | 15 | 102 |
| Chr14 | 157 | 13 | 17 | 17 | 204 |
| Chr15 | 46 | 10 | 5 | 10 | 71 |
| Chr16 | 71 | 0 | 23 | 8 | 102 |
| Chr17 | 59 | 8 | 32 | 12 | 111 |
| Chr18 | 31 | 4 | 15 | 9 | 59 |
| Chr19 | 147 | 3 | 10 | 34 | 194 |
| Chr20 | 29 | 1 | 81 | 8 | 119 |
| Chr21 | 18 | 24 | 3 | 4 | 49 |
| Chr22 | 60 | 15 | 19 | 11 | 105 |
| **Total** | **1,783** | **222** | **499** | **370** | **2,874** |

mCAs, mosaic chromosomal alterations; Chr, chromosome; CN-LOH, copy-neutral loss of heterozygosity

Supplementary Table 3: Counts of mCAs and expanded mCAs by event type of all carriers.

|  | **No. events** | **No. of expanded mCAs  (%)** |
| --- | --- | --- |
| **All autosomal mCAs** | 2,612 | 1,364 (52.2) |
| **CN-LOH** | 1,715 | 926 (54.0) |
| **Gain** | 197 | 91 (46.2) |
| **Loss** | 423 | 362 (85.6) |
| **Undetermined** | 365 | 22 (6.0) |

mCAs, mosaic chromosomal alterations; CN-LOH, copy-neutral loss of heterozygosity

Supplementary Table 4: Associations of all autosomal mCAs and mCAs subtypes with bladder cancer after excluding the bladder cancer identified in the first two years of follow-up.

|  | **Incident cases/N** | **Incidence rate（1/100,000)** | **Model1** | **Model2** | **Model3** |
| --- | --- | --- | --- | --- | --- |
| All autosomal mCAs - | 134/97,252 | 11.9 | 1.00 | 1.00 | 1.00 |
| All autosomal mCAs + | 11/2,611 | 37.7 | 2.62 (1.41, 4.86) | 2.62 (1.41, 4.87) | 2.62 (1.41, 4.87) |
| Non-expanded | 5/1,269 | 35.7 | 2.41 (0.98, 5.91) | 2.42 (0.99, 5.94) | 2.40 (0.98, 5.90) |
| Expanded | 6/1,342 | 39.5 | 2.82 (1.24, 6.42) | 2.82 (1.24, 6.41) | 2.83 (1.24, 6.45) |
| **mCAs subtypes** |  |  |  |  |  |
| Loss - | 139/99,440 | 12.1 | 1.00 | 1.00 | 1.00 |
| Loss + | 6/423 | 133.6 | 7.51 (3.29, 17.15) | 7.47 (3.26, 17.12) | 7.34 (3.19, 16.87) |
| Non-expanded | 1/61 | 148.9 | 8.72 (1.20, 63.24) | 9.08 (1.25, 66.02) | 9.99 (1.37, 73.02) |
| Expanded | 5/362 | 130.9 | 7.31 (2.97, 17.99) | 7.21 (2.92, 17.83) | 6.96 (2.80, 17.28) |
| CN-LOH - | 141/98,149 | 12.5 | 1.00 | 1.00 | 1.00 |
| CN-LOH + | 4/1,714 | 20.7 | 1.52 (0.56, 4.12) | 1.52 (0.56, 4.12) | 1.55 (0.57, 4.19) |
| Non-expanded | 3/788 | 34.1 | 2.39 (0.76, 7.54) | 2.38 (0.76, 7.51) | 2.36 (0.75, 7.44) |
| Expanded | 1/926 | 9.5 | 0.73 (0.10, 5.21) | 0.73 (0.10, 5.22) | 0.76 (0.11, 5.46) |

There were no bladder cancer cases among mosaic gain carriers. All multivariate models were stratified by age in the 5-year interval and 10 regions, and adjusted for sex and genotyping array (model 1), top 5 principal components, family history of cancer, and highest education (model 2), tobacco smoking, alcohol consumption, tea drinking, BMI, and PA (model 3).

HR, hazard ratios; CI, confidence interval; mCAs, mosaic chromosomal alterations; CN-LOH, copy-neutral loss of heterozygosity; BMI, body mass index; PA, physical activity

Supplementary Table 5: The HRs and 95%CIs of bladder cancer according to joint categories of level of physical activity and autosomal mCAs or mosaic loss.

| **mCAs and PA status** | **N** | **HR (95%CI）** |
| --- | --- | --- |
| **Autosomal mCAs** |  |  |
| High PA & Autosomal mCAs - | 73,507 | 1.00 |
| High PA & Autosomal mCAs + | 1,859 | 1.35 (0.50, 3.68) |
| Low PA & Autosomal mCAs - | 23,758 | 0.91 (0.62, 1.33) |
| Low PA & Autosomal mCAs + | 753 | 4.43 (2.10, 9.36) |
| **Loss** |  |  |
| High PA & Loss - | 75,074 | 1.00 |
| High PA & Loss + | 292 | 1.61 (0.22, 1.17) |
| Low PA & Loss - | 24,380 | 0.93 (0.64, 1.35) |
| Low PA & Loss + | 131 | 16.12 (6.34, 40.98) |

Adjusted covariates in the models were consistent with model 3 in Table 2, as appropriate.

HR, hazard ratios; CI, confidence interval; PA, physical activity; mCAs, mosaic chromosomal alteration

Supplementary Figure 1：Association between age and the status of autosomal mCAs by sex among CKB population.


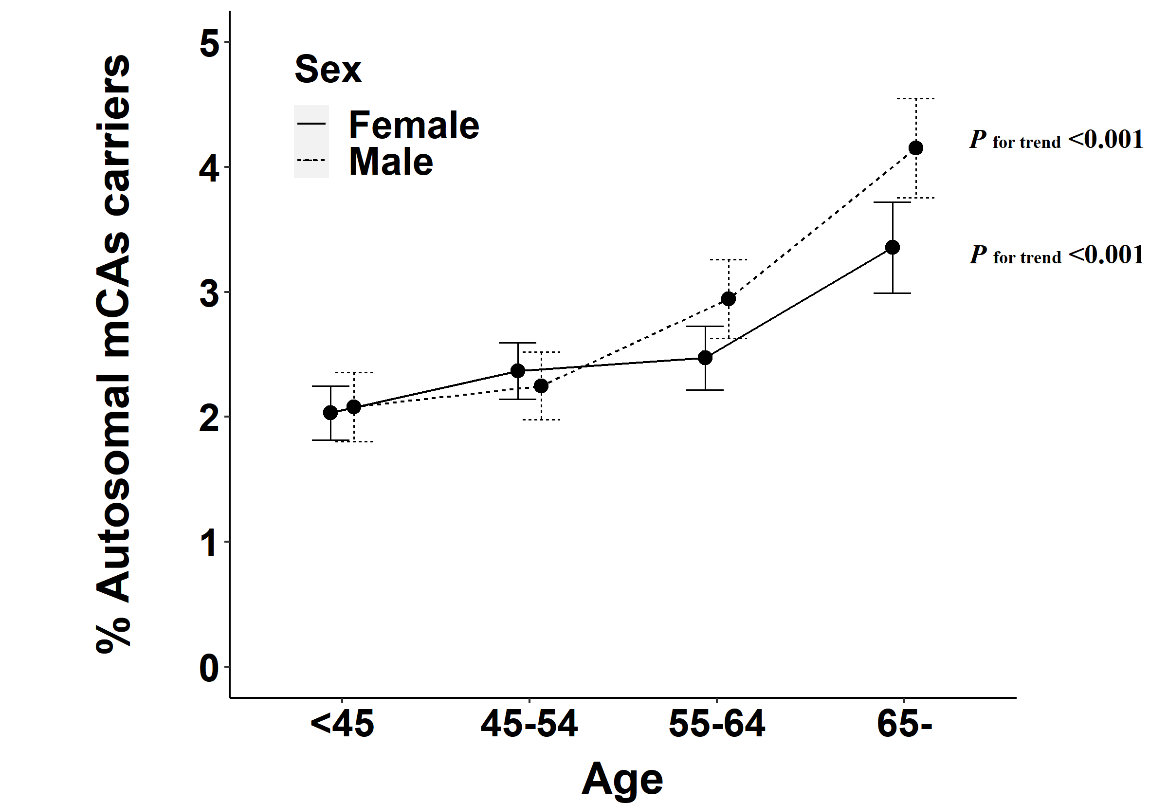


Tests for linear trend were conducted in both sexes by including each of the age groups in the logistic regression models as continuous variables.

mCAs, mosaic chromosomal alterations; CKB, China Kadoorie Biobank


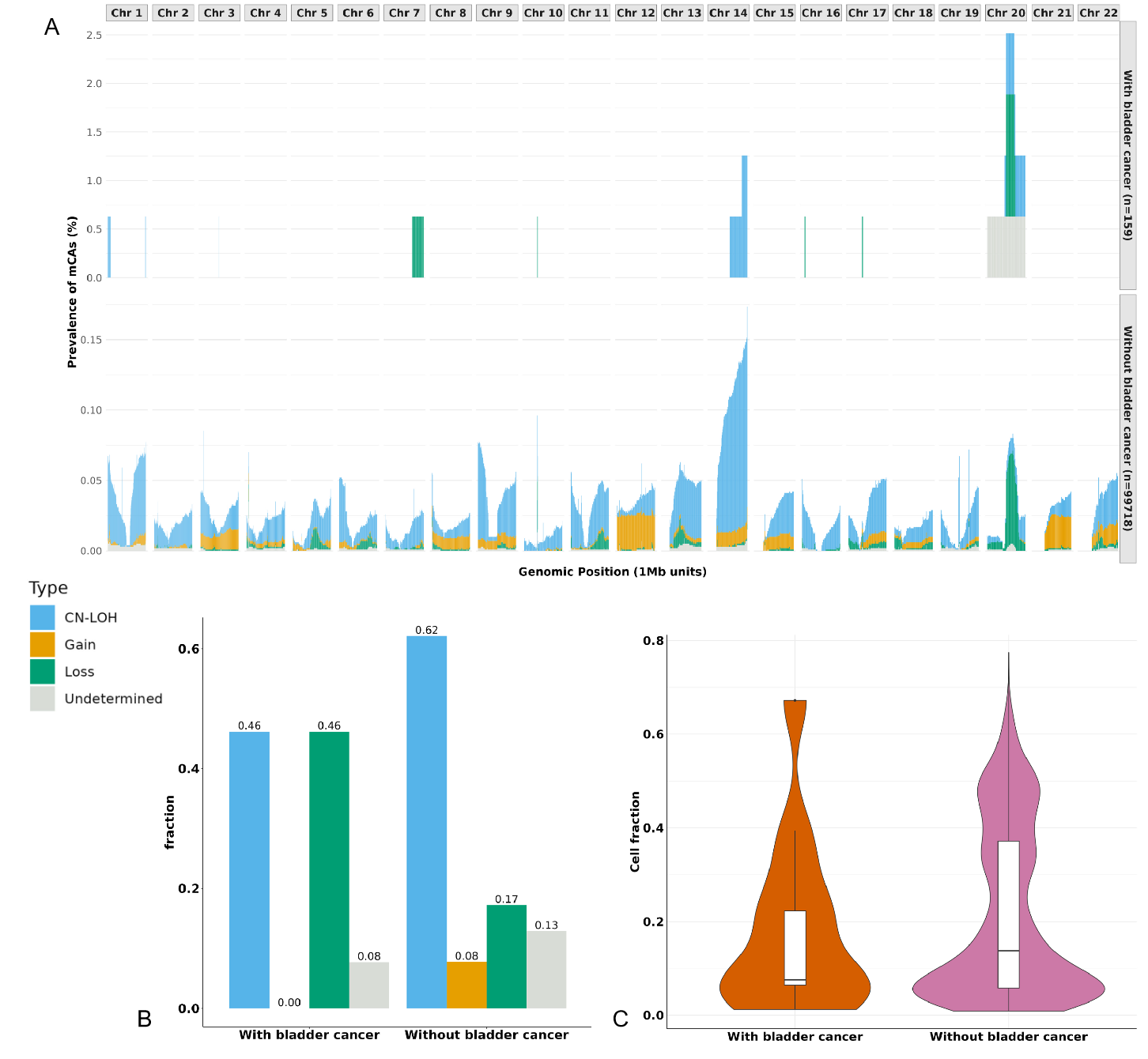
Supplementary Figure 2：Distribution of the nature of mCAs in the participants with and without bladder cancer.

1. Genomic distributions of four mCAs subtypes across 22 autosomal chromosomes among individuals with (above) and without (below) bladder cancer. The X axis was shown in 1 Mb units for each chromosome and the Y axis was the prevalence of mCAs events in the corresponding genomic window. The number of mCAs events detected in participants with and without bladder cancer was 13 and 2861, respectively.
2. Fractions of four mCAs subtypes among autosomal mCAs carriers with (left) and without (right) bladder cancer. The *P*-values for Fisher's exact test (two-sided) for each subtype comparison between two groups were as follows: 0.02 for loss, 0.26 for CN-LOH, 0.62 for gain, and 1.00 for undetermined.
3. Violin plots of the distribution of the estimated mCAs cell fraction among autosomal mCAs carriers with (left) and without (right) bladder cancer. The cell fraction of a total of 279 undetermined mCAs events could not be estimated due to the algorithmic issues.

mCAs, mosaic chromosomal alterations; CN-LOH, copy-neutral loss of heterozygosity
